# For robust big data analyses: a collection of 150 important pro-metastatic genes

**DOI:** 10.1186/s40880-016-0178-z

**Published:** 2017-01-21

**Authors:** Yan Mei, Jun-Ping Yang, Chao-Nan Qian

**Affiliations:** 1 State Key Laboratory of Oncology in South China, Collaborative Innovation Center for Cancer Medicine, Sun Yat-sen University Cancer Center, Guangzhou, 510060 Guangdong P. R. China; 2 Department of Nasopharyngeal Carcinoma, Sun Yat-sen University Cancer Center, Guangzhou, 510060 Guangdong P. R. China

**Keywords:** Pro-metastatic gene, Big data analysis, Renal cancer, Liver cancer

## Abstract

Metastasis is the greatest contributor to cancer-related death. In the era of precision medicine, it is essential to predict and to prevent the spread of cancer cells to significantly improve patient survival. Thanks to the application of a variety of high-throughput technologies, accumulating big data enables researchers and clinicians to identify aggressive tumors as well as patients with a high risk of cancer metastasis. However, there have been few large-scale gene collection studies to enable metastasis-related analyses. In the last several years, emerging efforts have identified pro-metastatic genes in a variety of cancers, providing us the ability to generate a pro-metastatic gene cluster for big data analyses. We carefully selected 285 genes with in vivo evidence of promoting metastasis reported in the literature. These genes have been investigated in different tumor types. We used two datasets downloaded from The Cancer Genome Atlas database, specifically, datasets of clear cell renal cell carcinoma and hepatocellular carcinoma, for validation tests, and excluded any genes for which elevated expression level correlated with longer overall survival in any of the datasets. Ultimately, 150 pro-metastatic genes remained in our analyses. We believe this collection of pro-metastatic genes will be helpful for big data analyses, and eventually will accelerate anti-metastasis research and clinical intervention.

## Background

Cancer metastasis is the greatest cause of death in almost all types of malignancies [1]. Multiple factors from the tumor and the host contribute to the formation and progression of distant secondary tumors [1, 2], and most of the mechanistic studies to date have mainly focused on the metastatic potential of tumor cells. It is believed that the metastasis of single cancer cells begins with the cells gaining the ability to migrate and invade. The cancer cells can gain motility in several ways, including epithelial-mesenchymal transition (EMT) and fusion of cancer cells to highly mobile bone marrow-derived cells [3, 4]. In the metastases formed by clusters of tumor cells, EMT may not be necessary [5]; however, the layer of endothelial cells enveloping the entire tumor cluster/embolus seems critical for the survival of tumor clusters [6].

The ability to identify cancer patients with a high risk of metastasis is essential in the era of precision medicine. In addition to applying clinicopathologic parameter combination, also known as clinical prognostic classifiers in some circumstances, molecular profiling based on high-throughput technologies is expected to allow for a more accurate and robust prognostic prediction of metastatic potential in patients. How to effectively analyze big data generated from high-throughput screening is an emerging issue for many bioinformaticians. We hypothesize that, with optimal weighting on the impact of each individual gene, a collection of key pro-metastatic genes could be useful to generate a prognostic tool to identify the metastatic potential of a specific tumor and novel signaling pathways underlying metastasis.

## Main text

The increased investigation of cancer metastasis in recent years has identified over 200 pro-metastatic genes. In this review, we aim to identify a group of key pro-metastatic genes with in vivo functional evidence and reasonable clinical relevance for application to big data analyses.

**Fig. 1:**
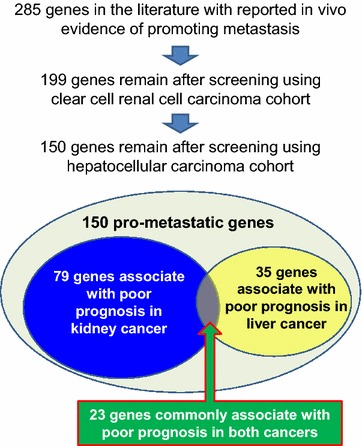
A schematic illustration of the study design and findings

Figure 1 summarizes the analytic procedure of this review. First, we carefully selected 285 genes from the literature through searching PubMed based on the following criteria: (1) author-provided evidence of promoting migration and/or invasion of cancer cells; (2) author-provided evidence of promoting metastasis in vivo using animal models; (3) when a gene has been reported as pro-metastatic in several articles, all articles reporting the link were reviewed, and the most convincing studies are listed as the key references in Table 1. In addition, we applied survival analyses as validation tests using the publicly available TCGA datasets (threshold = 0.05). For analyses of clear cell renal cell carcinoma (ccRCC), the mRNA expression data of 72 non-cancerous kidney tissues and 539 tumors [clear cell kidney carcinoma (KIRC) in the TCGA database] were downloaded. For analyses of hepatocellular carcinoma (HCC), the mRNA expression data of 50 non-cancerous liver tissues and 374 tumors [liver hepatocellular carcinoma (LIHC) in the TCGA database] were used. Normalization was performed using the DESeq method (Version 1.26.0). For each individual gene, the median expression level was used as a cut-off value to separate the patients into high and low expression groups. Genes were excluded if their elevated expression significantly associated with better patient prognosis in any patient cohort. Finally, 150 genes passed the tests and are listed in Table 1. Among them, 79 genes have significant prognostic values in the ccRCC patient cohort, 35 genes have significant prognostic values in the HCC cohort, and 23 genes have significant prognostic values in both cohorts.

**Table 1:** The list of 150 pro-metastatic genes with clinical relevance and key references

Number	Gene name	Clinical relevance validation (*P* value of overall survival analysis)		Reference
		ccRCC cohort	HCC cohort	
1	*ADAM9*	NS	0.001	[10]
2	*ADORA2B*	0.006	NS	[11]
3	*AGR2*	<0.001	NS	[12]
4	*AKT1*	NS	NS	[13]
5	*ANXA1*	NS	NS	[14]
6	*APOBEC3G*	0.045	NS	[15]
7	*ATF4*	0.001	0.031	[16]
8	*AXL*	0.005	NS	[17]
9	*BACH1*	NS	NS	[18]
10	*BCL2L1*	NS	NS	[19]
11	*BCL3*	<0.001	NS	[20]
12	*BIRC5*	<0.001	<0.001	[21]
13	*BSG*	NS	0.004	[22]
14	*C5AR1*	NS	NS	[23]
15	*CAV1*	NS	NS	[24]
16	*CCL2*	NS	NS	[25]
17	*CCR7*	NS	0.002	[26]
18	*CD24*	NS	NS	[27]
19	*CD44*	0.016	NS	[28]
20	*CDCP1*	NS	NS	[29]
21	*CEACAM6*	0.004	NS	[30]
22	*CEBPD*	0.022	NS	[31]
23	*CENPF*	<0.001	0.008	[32]
24	*CHD1L*	<0.001	0.007	[33]
25	*CHI3L1*	NS	NS	[34]
26	*CLDN9*	0.039	NS	[35]
27	*COL6A1*	<0.001	NS	[36]
28	*COMP*	0.040	NS	[37]
29	*CSNK2A2*	NS	NS	[38]
30	*CTSB*	NS	NS	[38]
31	*CTSZ*	<0.001	NS	[39]
32	*CXCL1*	<0.001	0.001	[40]
33	*CXCL10*	NS	NS	[41]
34	*CXCL8*	0.002	<0.001	[42]
35	*CXCR4*	NS	NS	[43]
36	*E2F1*	0.001	0.005	[44]
37	*EIF5A*	<0.001	NS	[45]
38	*ELF5*	NS	NS	[46]
39	*ENAH*	NS	0.012	[47]
40	*ENPP2*	NS	NS	[48]
41	*ETV4*	0.003	0.001	[49]
42	*EZH2*	<0.001	<0.001	[50]
43	*FGFR1*	NS	NS	[51]
44	*FLOT2*	NS	NS	[52]
45	*FOSL1*	<0.001	NS	[53]
46	*FOXC1*	NS	NS	[54]
47	*FOXM1*	<0.001	0.009	[55]
48	*FOXQ1*	NS	NS	[56]
49	*FZD2*	<0.001	NS	[57]
50	*GABRA3*	NS	0.004	[58]
51	*GDF15*	NS	NS	[59]
52	*GHRL*	<0.001	NS	[60]
53	*GLI2*	<0.001	NS	[61]
54	*GOLM1*	NS	0.049	[62]
55	*GRK3*	NS	NS	[63]
56	*HMGB1*	NS	NS	[64]
57	*HMMR*	0.003	<0.001	[65]
58	*HOXB13*	<0.001	NS	[66]
59	*HOXB7*	NS	NS	[67]
60	*HOXB9*	<0.001	NS	[68]
61	*ID1*	NS	NS	[69]
62	*IDO1*	NS	NS	[70]
63	*IGFBP2*	NS	NS	[71]
64	*IL32*	NS	NS	[72]
65	*IL5*	NS	NS	[73]
66	*IL6*	<0.001	NS	[74]
67	*IP6K2*	0.001	NS	[75]
68	*ITGA3*	NS	NS	[76]
69	*ITGA5*	0.018	0.011	[77]
70	*ITGBL1*	NS	NS	[78]
71	*KISS1R*	NS	NS	[79]
72	*KLF8*	NS	NS	[80]
73	*L1CAM*	0.007	NS	[81]
74	*LAMB3*	0.001	NS	[67]
75	*LEF1*	0.007	NS	[82]
76	*LGALS1*	<0.001	0.048	[83]
77	*LGALS3*	NS	NS	[84]
78	*LOX*	NS	0.047	[85]
79	*LOXL2*	0.033	NS	[86]
80	*MBD4*	NS	NS	[87]
81	*MCAM*	NS	NS	[88]
82	*MET*	NS	NS	[89]
83	*MMP1*	0.030	0.002	[90]
84	*MMP16*	NS	NS	[91]
85	*MMP9*	0.001	0.009	[92]
86	*MTA1*	0.015	NS	[93]
87	*MTA2*	0.001	NS	[94]
88	*MYB*	0.031	0.021	[95]
89	*NFATC2*	NS	NS	[96]
90	*NRP2*	NS	NS	[97]
91	*NTRK3*	NS	0.044	[98]
92	*PARP1*	NS	NS	[99]
93	*PCDH7*	NS	NS	[100]
94	*PDGFRB*	NS	NS	[101]
95	*PDPN*	0.034	NS	[102]
96	*PELP1*	0.011	NS	[103]
97	*PHGDH*	NS	NS	[104]
98	*PHIP*	NS	NS	[105]
99	*PLAUR*	<0.001	NS	[35]
100	*PLOD2*	0.004	0.008	[106]
101	*POSTN*	NS	NS	[107]
102	*PPIA*	0.015	0.038	[108]
103	*PRRX1*	0.045	NS	[109]
104	*PRSS50*	<0.001	NS	[89]
105	*PTGS2*	0.040	NS	[110]
106	*PTTG1*	<0.001	0.004	[111]
107	*PXN*	0.001	NS	[112]
108	*RAB22A*	0.024	NS	[113]
109	*RAC1*	NS	NS	[97]
110	*RAF1*	0.025	NS	[23]
111	*RHOC*	0.030	NS	[114]
112	*ROR2*	0.001	NS	[115]
113	*RRAS*	<0.001	NS	[116]
114	*RUNX3*	NS	0.032	[117]
115	*S100A4*	NS	NS	[118]
116	*S100P*	NS	NS	[119]
117	*SEMA3E*	<0.001	NS	[120]
118	*SFRP2*	0.020	NS	[121]
119	*SIX2*	0.001	0.036	[122]
120	*SNAI1*	0.045	NS	[123]
121	*SNAI2*	NS	NS	[124]
122	*SOX12*	<0.001	0.045	[125]
123	*SOX4*	NS	0.018	[126]
124	*SPINK1*	<0.001	NS	[127]
125	*SPON2*	<0.001	NS	[128]
126	*SPP1*	NS	0.000	[129]
127	*SRC*	<0.001	0.037	[130]
128	*SRGN*	NS	NS	[131]
129	*SRPK1*	NS	NS	[132]
130	*TACSTD2*	NS	NS	[133]
131	*TDO2*	0.020	NS	[134]
132	*TF*	<0.001	NS	[135]
133	*TGFB1*	0.008	NS	[73]
134	*TGM2*	0.003	NS	[136]
135	*TNC*	NS	NS	[137]
136	*TNFSF10*	NS	NS	[138]
137	*TNK2*	0.016	NS	[139]
138	*TP73*	0.016	NS	[140]
139	*TPO*	0.043	NS	[141]
140	*TRIM28*	NS	0.00	[142]
141	*TWIST1*	0.002	NS	[143]
142	*UBE2* *N*	NS	NS	[144]
143	*VAV1*	0.038	NS	[145]
144	*VEGFB*	NS	NS	[146]
145	*VIM*	0.014	NS	[147]
146	*WASF3*	NS	NS	[148]
147	*WNT5A*	0.008	NS	[149]
148	*WSB1*	<0.001	NS	[150]
149	*YBX1*	0.038	<0.001	[151]
150	*ZEB2*	NS	NS	[152]

*NS* not significant

**Fig. 2:**
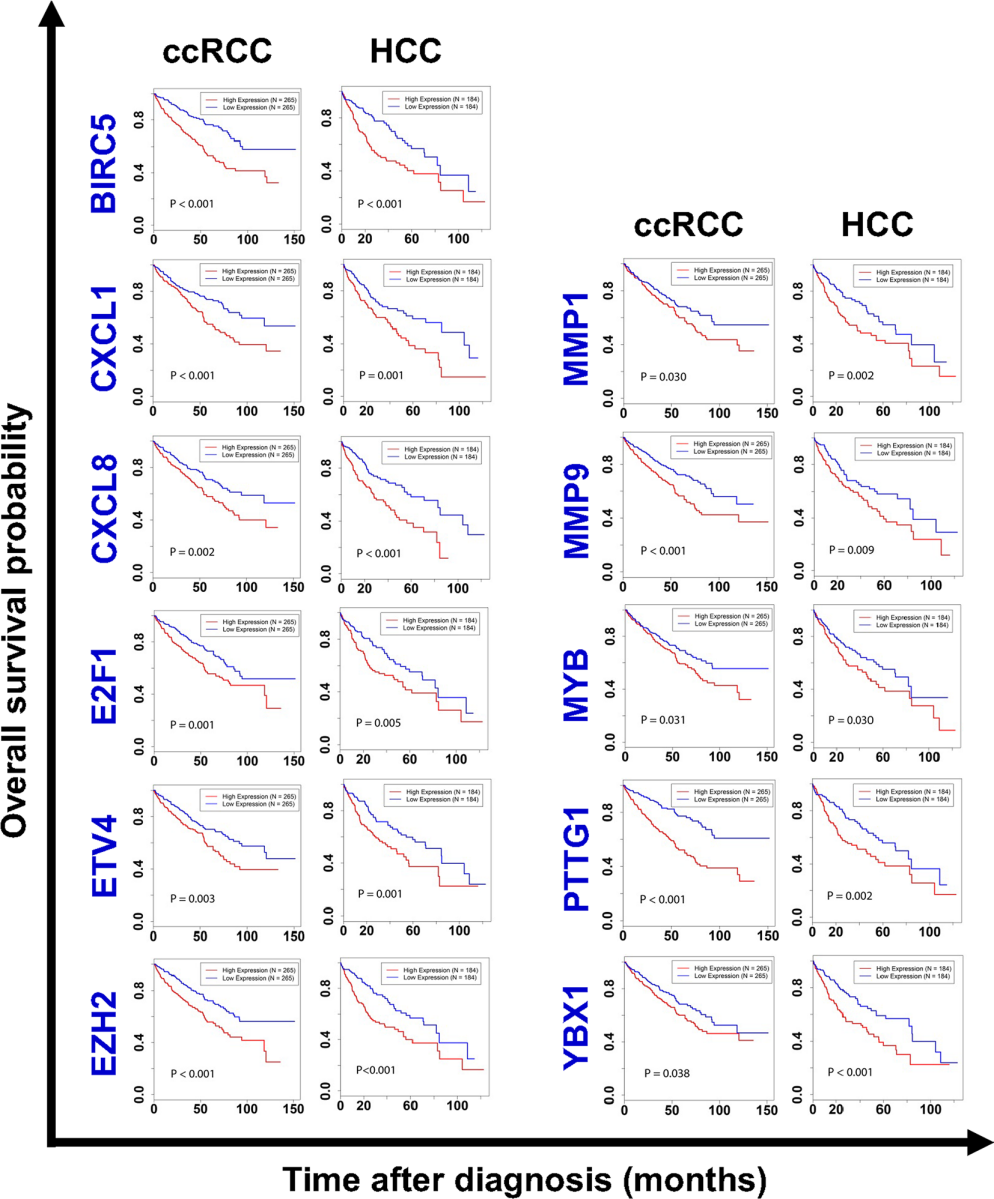
The survival curves of two cohorts of cancer patients comparing the mRNA expression levels of 11 genes. The data were retrieved from The Cancer Genome Atlas (TCGA) database. The survival curves were plotted using the Kaplan–Meier method and compared using the log-rank test. Consistently, among all 11 genes presented in this figure, elevated gene expression levels significantly associate with shorter overall patient survival (*P* < 0.05) in both tumor types. *ccRCC* clear cell renal cell carcinoma, *HCC* hepatocellular carcinoma

Although different tumor types are believed to rely on different molecular mechanisms for metastasis, 23 common pro-metastatic genes have been identified in our analyses, associating with poor prognosis in both cancer types. Among them, we are most interested in 11 genes that are not only statistically significant in terms of prognostic impact but also associated with distinct overall survival curves in both cohorts, suggesting the genes’ profound biological impacts on tumor progression. For the other 12 genes, although their biological impact on tumor progression were found to be significant in log-rank tests in both cohorts, the survival curves of high versus low expression groups crossed at some time points. The 11 most interesting genes are *BIRC5 (Survivin), CXCL1, CXCL8 (IL8), E2F1, ETV4, EZH2, MMP1, MMP9, MYB, PTTG1, and YBX1*. Figure 2 shows the survival curves of patients with either ccRCC or HCC expressing these 11 genes. Our findings suggest that different tumor types may partially share some common metastatic mechanisms, therefore strengthening the rationale of applying the list of 150 pro-metastatic genes to big data analyses. Interestingly, 4 of these 11 genes encode secreted proteins, namely, *CXCL1*, *CXCL8*, *MMP1*, and *MMP9*, which are ideal pharmaceutical targets for blocking cancer metastasis.

Although not covered in this review article, emerging data regarding the regulatory roles of non-coding RNA in metastasis have linked different pro-metastatic genes to forming signaling cascades [7–9]. Further investigation into the roles of non-coding RNA in metastasis is warranted.

## Conclusions

In summary, we present here a collection of 150 important pro-metastatic genes for big data analyses. We expect more key molecules to be identified and validated in the near future to be included in the list, thereby accelerating the efforts in preventing and treating cancer metastasis.

## References

[1] Steeg PS (2016). Targeting metastasis. Nat Rev Cancer.

[2] Li W, Shen LJ, Chen T, Sun XQ, Zhang Y, Wu M (2016). Overweight/obese status associates with favorable outcome in patients with metastatic nasopharyngeal carcinoma: a 10-year retrospective study. Chin J Cancer..

[3] Pawelek JM (2014). Fusion of bone marrow-derived cells with cancer cells: metastasis as a secondary disease in cancer. Chin J Cancer..

[4] Savagner P (2015). Epithelial-mesenchymal transitions: from cell plasticity to concept elasticity. Curr Top Dev Biol.

[5] Seton-Rogers S (2016). Epithelial-mesenchymal transition: untangling EMT’s functions. Nat Rev Cancer.

[6] Ding T, Xu J, Zhang Y, Guo RP, Wu WC, Zhang SD (2011). Endothelium-coated tumor clusters are associated with poor prognosis and micrometastasis of hepatocellular carcinoma after resection. Cancer.

[7] Jiang C, Li X, Zhao H, Liu H (2016). Long non-coding RNAs: potential new biomarkers for predicting tumor invasion and metastasis. Mol Cancer..

[8] Sun R, Qin C, Jiang B, Fang S, Pan X, Peng L (2016). Down-regulation of MALAT1 inhibits cervical cancer cell invasion and metastasis by inhibition of epithelial-mesenchymal transition. Mol BioSyst.

[9] Sun Y, Guo F, Bagnoli M, Xue FX, Sun BC, Shmulevich I (2015). Key nodes of a microRNA network associated with the integrated mesenchymal subtype of high-grade serous ovarian cancer. Chin J Cancer..

[10] Lin CY, Chen HJ, Huang CC, Lai LC, Lu TP, Tseng GC (2014). ADAM9 promotes lung cancer metastases to brain by a plasminogen activator-based pathway. Cancer Res.

[11] Mittal D, Sinha D, Barkauskas D, Young A, Kalimutho M, Stannard K (2016). Adenosine 2B receptor expression on cancer cells promotes metastasis. Cancer Res.

[12] Dumartin L, Whiteman HJ, Weeks ME, Hariharan D, Dmitrovic B, Iacobuzio-Donahue CA (2011). AGR2 is a novel surface antigen that promotes the dissemination of pancreatic cancer cells through regulation of cathepsins B and D. Cancer Res.

[13] Cho JH, Robinson JP, Arave RA, Burnett WJ, Kircher DA, Chen G (2015). AKT1 activation promotes development of melanoma metastases. Cell Rep..

[14] de Graauw M, van Miltenburg MH, Schmidt MK, Pont C, Lalai R, Kartopawiro J (2010). Annexin A1 regulates TGF-beta signaling and promotes metastasis formation of basal-like breast cancer cells. Proc Natl Acad Sci USA.

[15] Ding Q, Chang CJ, Xie X, Xia W, Yang JY, Wang SC (2011). APOBEC3G promotes liver metastasis in an orthotopic mouse model of colorectal cancer and predicts human hepatic metastasis. J Clin Investig.

[16] Dey S, Sayers CM, Verginadis II, Lehman SL, Cheng Y, Cerniglia GJ (2015). ATF4-dependent induction of heme oxygenase 1 prevents anoikis and promotes metastasis. J Clin Investig.

[17] Rankin EB, Fuh KC, Castellini L, Viswanathan K, Finger EC, Diep AN (2014). Direct regulation of GAS6/AXL signaling by HIF promotes renal metastasis through SRC and MET. Proc Natl Acad Sci USA.

[18] Yun J, Frankenberger CA, Kuo WL, Boelens MC, Eves EM, Cheng N (2011). Signalling pathway for RKIP and Let-7 regulates and predicts metastatic breast cancer. EMBO J.

[19] Choi S, Chen Z, Tang LH, Fang Y, Shin SJ, Panarelli NC (2016). Bcl-xL promotes metastasis independent of its anti-apoptotic activity. Nat Commun..

[20] Wakefield A, Soukupova J, Montagne A, Ranger J, French R, Muller WJ (2013). Bcl3 selectively promotes metastasis of ERBB2-driven mammary tumors. Cancer Res.

[21] McKenzie JA, Liu T, Jung JY, Jones BB, Ekiz HA, Welm AL (2013). Survivin promotion of melanoma metastasis requires upregulation of alpha5 integrin. Carcinogenesis.

[22] Kong LM, Liao CG, Zhang Y, Xu J, Li Y, Huang W (2014). A regulatory loop involving miR-22, Sp1, and c-Myc modulates CD147 expression in breast cancer invasion and metastasis. Cancer Res.

[23] Vadrevu SK, Chintala NK, Sharma SK, Sharma P, Cleveland C, Riediger L (2014). Complement c5a receptor facilitates cancer metastasis by altering T-cell responses in the metastatic niche. Cancer Res.

[24] Thomas S, Overdevest JB, Nitz MD, Williams PD, Owens CR, Sanchez-Carbayo M (2011). Src and caveolin-1 reciprocally regulate metastasis via a common downstream signaling pathway in bladder cancer. Cancer Res.

[25] Bonapace L, Coissieux MM, Wyckoff J, Mertz KD, Varga Z, Junt T (2014). Cessation of CCL2 inhibition accelerates breast cancer metastasis by promoting angiogenesis. Nature.

[26] Yu S, Duan J, Zhou Z, Pang Q, Wuyang J, Liu T (2008). A critical role of CCR7 in invasiveness and metastasis of SW620 colon cancer cell in vitro and in vivo. Cancer Biol Ther.

[27] Overdevest JB, Knubel KH, Duex JE, Thomas S, Nitz MD, Harding MA (2012). CD24 expression is important in male urothelial tumorigenesis and metastasis in mice and is androgen regulated. Proc Natl Acad Sci USA.

[28] Su J, Wu S, Wu H, Li L, Guo T (2015). CD44 is functionally crucial for driving lung cancer stem cells metastasis through Wnt/beta-catenin-FoxM1-Twist signaling. Mol Carcinog.

[29] Liu H, Ong SE, Badu-Nkansah K, Schindler J, White FM, Hynes RO (2011). CUB-domain-containing protein 1 (CDCP1) activates Src to promote melanoma metastasis. Proc Natl Acad Sci USA.

[30] Blumenthal RD, Hansen HJ, Goldenberg DM (2005). Inhibition of adhesion, invasion, and metastasis by antibodies targeting CEACAM6 (NCA-90) and CEACAM5 (Carcinoembryonic Antigen). Cancer Res.

[31] Balamurugan K, Wang JM, Tsai HH, Sharan S, Anver M, Leighty R (2010). The tumour suppressor C/EBPdelta inhibits FBXW7 expression and promotes mammary tumour metastasis. EMBO J.

[32] Lin SC, Kao CY, Lee HJ, Creighton CJ, Ittmann MM, Tsai SJ (2016). Dysregulation of miRNAs-COUP-TFII-FOXM1-CENPF axis contributes to the metastasis of prostate cancer. Nat Commun..

[33] Chen L, Chan TH, Yuan YF, Hu L, Huang J, Ma S (2010). CHD1L promotes hepatocellular carcinoma progression and metastasis in mice and is associated with these processes in human patients. J Clin Investig.

[34] Ma B, Herzog EL, Lee CG, Peng X, Lee CM, Chen X (2015). Role of chitinase 3-like-1 and semaphorin 7a in pulmonary melanoma metastasis. Cancer Res.

[35] Sharma RK, Chheda ZS, Das Purkayastha BP, Gomez-Gutierrez JG, Jala VR, Haribabu B (2016). A spontaneous metastasis model reveals the significance of claudin-9 overexpression in lung cancer metastasis. Clin Exp Metastasis.

[36] Blanco MA, LeRoy G, Khan Z, Aleckovic M, Zee BM, Garcia BA (2012). Global secretome analysis identifies novel mediators of bone metastasis. Cell Res.

[37] Englund E, Bartoschek M, Reitsma B, Jacobsson L, Escudero-Esparza A, Orimo A (2016). Cartilage oligomeric matrix protein contributes to the development and metastasis of breast cancer. Oncogene.

[38] Liu Y, Amin EB, Mayo MW, Chudgar NP, Bucciarelli PR, Kadota K (2016). CK2alpha’ drives lung cancer metastasis by targeting BRMS1 nuclear export and degradation. Cancer Res.

[39] Sevenich L, Schurigt U, Sachse K, Gajda M, Werner F, Muller S (2010). Synergistic antitumor effects of combined cathepsin B and cathepsin Z deficiencies on breast cancer progression and metastasis in mice. Proc Natl Acad Sci USA.

[40] Acharyya S, Oskarsson T, Vanharanta S, Malladi S, Kim J, Morris PG (2012). A CXCL1 paracrine network links cancer chemoresistance and metastasis. Cell.

[41] Lee JH, Kim HN, Kim KO, Jin WJ, Lee S, Kim HH (2012). CXCL10 promotes osteolytic bone metastasis by enhancing cancer outgrowth and osteoclastogenesis. Cancer Res.

[42] Li XJ, Peng LX, Shao JY, Lu WH, Zhang JX, Chen S (2012). As an independent unfavorable prognostic factor, IL-8 promotes metastasis of nasopharyngeal carcinoma through induction of epithelial-mesenchymal transition and activation of AKT signaling. Carcinogenesis.

[43] Choi YH, Burdick MD, Strieter BA, Mehrad B, Strieter RM (2014). CXCR4, but not CXCR7, discriminates metastatic behavior in non-small cell lung cancer cells. Mol Cancer Res.

[44] Rennhack J, Andrechek E (2015). Conserved E2F mediated metastasis in mouse models of breast cancer and HER2 positive patients. Oncoscience..

[45] Fujimura K, Choi S, Wyse M, Strnadel J, Wright T, Klemke R (2015). Eukaryotic translation initiation factor 5A (EIF5A) regulates pancreatic cancer metastasis by modulating RhoA and Rho-associated kinase (ROCK) protein expression levels. J Biol Chem.

[46] Gallego-Ortega D, Ledger A, Roden DL, Law AM, Magenau A, Kikhtyak Z (2015). ELF5 drives lung metastasis in luminal breast cancer through recruitment of Gr1 + CD11b + Myeloid-Derived suppressor cells. PLoS Biol.

[47] Santiago-Medina M, Yang J (2016). MENA promotes tumor-intrinsic metastasis through ECM remodeling and haptotaxis. Cancer Discov..

[48] Leblanc R, Lee SC, David M, Bordet JC, Norman DD, Patil R (2014). Interaction of platelet-derived autotaxin with tumor integrin alphaVbeta3 controls metastasis of breast cancer cells to bone. Blood.

[49] Aytes A, Mitrofanova A, Kinkade CW, Lefebvre C, Lei M, Phelan V (2013). ETV4 promotes metastasis in response to activation of PI3-kinase and Ras signaling in a mouse model of advanced prostate cancer. Proc Natl Acad Sci USA.

[50] Tong ZT, Cai MY, Wang XG, Kong LL, Mai SJ, Liu YH (2012). EZH2 supports nasopharyngeal carcinoma cell aggressiveness by forming a co-repressor complex with HDAC1/HDAC2 and snail to inhibit E-cadherin. Oncogene.

[51] Weekes D, Kashima TG, Zandueta C, Perurena N, Thomas DP, Sunters A (2016). Regulation of osteosarcoma cell lung metastasis by the c-Fos/AP-1 target FGFR1. Oncogene.

[52] Berger T, Ueda T, Arpaia E, Chio II, Shirdel EA, Jurisica I (2013). Flotillin-2 deficiency leads to reduced lung metastases in a mouse breast cancer model. Oncogene.

[53] Desmet CJ, Gallenne T, Prieur A, Reyal F, Visser NL, Wittner BS (2013). Identification of a pharmacologically tractable Fra-1/ADORA2B axis promoting breast cancer metastasis. Proc Natl Acad Sci USA.

[54] Xia L, Huang W, Tian D, Zhu H, Qi X, Chen Z (2013). Overexpression of forkhead box C1 promotes tumor metastasis and indicates poor prognosis in hepatocellular carcinoma. Hepatology.

[55] Xue J, Lin X, Chiu WT, Chen YH, Yu G, Liu M (2014). Sustained activation of SMAD3/SMAD4 by FOXM1 promotes TGF-beta-dependent cancer metastasis. J Clin Investig.

[56] Xia L, Huang W, Tian D, Zhang L, Qi X, Chen Z (2014). Forkhead box Q1 promotes hepatocellular carcinoma metastasis by transactivating ZEB2 and VersicanV1 expression. Hepatology.

[57] Gujral TS, Chan M, Peshkin L, Sorger PK, Kirschner MW, MacBeath G (2014). A noncanonical Frizzled2 pathway regulates epithelial-mesenchymal transition and metastasis. Cell.

[58] Gumireddy K, Li A, Kossenkov AV, Sakurai M, Yan J, Li Y (2016). The mRNA-edited form of GABRA3 suppresses GABRA3-mediated Akt activation and breast cancer metastasis. Nat Commun..

[59] Li C, Wang J, Kong J, Tang J, Wu Y, Xu E (2016). GDF15 promotes EMT and metastasis in colorectal cancer. Oncotarget..

[60] Lin TC, Liu YP, Chan YC, Su CY, Lin YF, Hsu SL (2015). Ghrelin promotes renal cell carcinoma metastasis via Snail activation and is associated with poor prognosis. J Pathol..

[61] Xu J, Acharya S, Sahin O, Zhang Q, Saito Y, Yao J (2015). 14-3-3zeta turns TGF-beta’s function from tumor suppressor to metastasis promoter in breast cancer by contextual changes of Smad partners from p53 to Gli2. Cancer Cell.

[62] Ye QH, Zhu WW, Zhang JB, Qin Y, Lu M, Lin GL (2016). GOLM1 modulates EGFR/RTK cell-surface recycling to drive hepatocellular carcinoma metastasis. Cancer Cell.

[63] Li W, Ai N, Wang S, Bhattacharya N, Vrbanac V, Collins M (2014). GRK3 is essential for metastatic cells and promotes prostate tumor progression. Proc Natl Acad Sci USA.

[64] Ni P, Zhang Y, Liu Y, Lin X, Su X, Lu H (2015). HMGB1 silence could promote MCF-7 cell apoptosis and inhibit invasion and metastasis. Int J Clin Exp Pathol..

[65] Du YC, Chou CK, Klimstra DS, Varmus H (2011). Receptor for hyaluronan-mediated motility isoform B promotes liver metastasis in a mouse model of multistep tumorigenesis and a tail vein assay for metastasis. Proc Natl Acad Sci USA.

[66] Kim YR, Kim IJ, Kang TW, Choi C, Kim KK, Kim MS (2014). HOXB13 downregulates intracellular zinc and increases NF-kappaB signaling to promote prostate cancer metastasis. Oncogene.

[67] Liu S, Jin K, Hui Y, Fu J, Jie C, Feng S (2015). HOXB7 promotes malignant progression by activating the TGFbeta signaling pathway. Cancer Res.

[68] Nguyen DX, Chiang AC, Zhang XH, Kim JY, Kris MG, Ladanyi M (2009). WNT/TCF signaling through LEF1 and HOXB9 mediates lung adenocarcinoma metastasis. Cell.

[69] Gumireddy K, Li A, Kossenkov AV, Cai KQ, Liu Q, Yan J (2014). ID1 promotes breast cancer metastasis by S100A9 regulation. Mol Cancer Res.

[70] Smith C, Chang MY, Parker KH, Beury DW, DuHadaway JB, Flick HE (2012). IDO is a nodal pathogenic driver of lung cancer and metastasis development. Cancer Discov..

[71] Gao S, Sun Y, Zhang X, Hu L, Liu Y, Chua YX (2016). IGFBP2 activates the NF-kappaB pathway to drive epithelial-mesenchymal transition and invasive character in pancreatic ductal adenocarcinoma. Cancer Res.

[72] Tsai CY, Wang CS, Tsai MM, Chi HC, Cheng WL, Tseng YH (2014). Interleukin-32 increases human gastric cancer cell invasion associated with tumor progression and metastasis. Clin Cancer Res.

[73] Zaynagetdinov R, Sherrill TP, Gleaves LA, McLoed AG, Saxon JA, Habermann AC (2015). Interleukin-5 facilitates lung metastasis by modulating the immune microenvironment. Cancer Res.

[74] Oh K, Moon HG, Lee DS, Yoo YB (2015). Tissue transglutaminase-interleukin-6 axis facilitates peritoneal tumor spreading and metastasis of human ovarian cancer cells. Lab Anim Res..

[75] Rao F, Xu J, Fu C, Cha JY, Gadalla MM, Xu R (2015). Inositol pyrophosphates promote tumor growth and metastasis by antagonizing liver kinase B1. Proc Natl Acad Sci USA.

[76] Zhou B, Gibson-Corley KN, Herndon ME, Sun Y, Gustafson-Wagner E, Teoh-Fitzgerald M (2014). Integrin alpha3beta1 can function to promote spontaneous metastasis and lung colonization of invasive breast carcinoma. Mol Cancer Res.

[77] Valastyan S, Chang A, Benaich N, Reinhardt F, Weinberg RA (2010). Concurrent suppression of integrin alpha5, radixin, and RhoA phenocopies the effects of miR-31 on metastasis. Cancer Res.

[78] Li XQ, Du X, Li DM, Kong PZ, Sun Y, Liu PF (2015). ITGBL1 is a Runx2 transcriptional target and promotes breast cancer bone metastasis by activating the TGFbeta signaling pathway. Cancer Res.

[79] Cho SG, Wang Y, Rodriguez M, Tan K, Zhang W, Luo J (2011). Haploinsufficiency in the prometastasis Kiss1 receptor Gpr54 delays breast tumor initiation, progression, and lung metastasis. Cancer Res.

[80] Lu H, Hu L, Yu L, Wang X, Urvalek AM, Li T (2014). KLF8 and FAK cooperatively enrich the active MMP14 on the cell surface required for the metastatic progression of breast cancer. Oncogene.

[81] Weinspach D, Seubert B, Schaten S, Honert K, Sebens S, Altevogt P (2014). Role of L1 cell adhesion molecule (L1CAM) in the metastatic cascade: promotion of dissemination, colonization, and metastatic growth. Clin Exp Metastasis.

[82] Wang XM, Li J, Yan MX, Liu L, Jia DS, Geng Q (2013). Integrative analyses identify osteopontin, LAMB3 and ITGB1 as critical pro-metastatic genes for lung cancer. PLoS ONE.

[83] Hsu YL, Wu CY, Hung JY, Lin YS, Huang MS, Kuo PL (2013). Galectin-1 promotes lung cancer tumor metastasis by potentiating integrin alpha6beta4 and Notch1/Jagged2 signaling pathway. Carcinogenesis.

[84] Braeuer RR, Zigler M, Kamiya T, Dobroff AS, Huang L, Choi W (2012). Galectin-3 contributes to melanoma growth and metastasis via regulation of NFAT1 and autotaxin. Cancer Res.

[85] Cox TR, Gartland A, Erler JT (2016). Lysyl oxidase, a targetable secreted molecule involved in cancer metastasis. Cancer Res.

[86] Barker HE, Chang J, Cox TR, Lang G, Bird D, Nicolau M (2011). LOXL2-mediated matrix remodeling in metastasis and mammary gland involution. Cancer Res.

[87] Cunha S, Lin YC, Goossen EA, DeVette CI, Albertella MR, Thomson S (2014). The RON receptor tyrosine kinase promotes metastasis by triggering MBD4-dependent DNA methylation reprogramming. Cell Rep..

[88] Wu GJ, Fu P, Wang SW, Wu MW (2008). Enforced expression of MCAM/MUC18 increases in vitro motility and invasiveness and in vivo metastasis of two mouse melanoma K1735 sublines in a syngeneic mouse model. Mol Cancer Res.

[89] Firon M, Shaharabany M, Altstock RT, Horev J, Abramovici A, Resau JH (2000). Dominant negative Met reduces tumorigenicity-metastasis and increases tubule formation in mammary cells. Oncogene.

[90] Wu K, Fukuda K, Xing F, Zhang Y, Sharma S, Liu Y (2015). Roles of the cyclooxygenase 2 matrix metalloproteinase 1 pathway in brain metastasis of breast cancer. J Biol Chem.

[91] Tatti O, Gucciardo E, Pekkonen P, Holopainen T, Louhimo R, Repo P (2015). MMP16 mediates a proteolytic switch to promote cell-cell adhesion, collagen alignment, and lymphatic invasion in melanoma. Cancer Res.

[92] Chen X, Su Y, Fingleton B, Acuff H, Matrisian LM, Zent R (2005). Increased plasma MMP9 in integrin alpha1-null mice enhances lung metastasis of colon carcinoma cells. Int J Cancer.

[93] Deng L, Yang H, Tang J, Lin Z, Yin A, Gao Y (2015). Inhibition of MTA1 by ERalpha contributes to protection hepatocellular carcinoma from tumor proliferation and metastasis. J Exp Clin Cancer Res..

[94] Zhang B, Zhang H, Shen G (2015). Metastasis-associated protein 2 (MTA2) promotes the metastasis of non-small-cell lung cancer through the inhibition of the cell adhesion molecule Ep-CAM and E-cadherin. Jpn J Clin Oncol.

[95] Li Y, Jin K, van Pelt GW, van Dam H, Yu X, Mesker WE (2016). c-Myb enhances breast cancer invasion and metastasis through the Wnt/beta-Catenin/Axin2 pathway. Cancer Res.

[96] Shoshan E, Braeuer RR, Kamiya T, Mobley AK, Huang L, Vasquez ME (2016). NFAT1 directly regulates IL8 and MMP3 to promote melanoma tumor growth and metastasis. Cancer Res.

[97] Fung TM, Ng KY, Tong M, Chen JN, Chai S, Chan KT (2016). Neuropilin-2 promotes tumourigenicity and metastasis in oesophageal squamous cell carcinoma through ERK-MAPK-ETV4-MMP-E-cadherin deregulation. J Pathol..

[98] Faltermeier CM, Drake JM, Clark PM, Smith BA, Zong Y, Volpe C (2016). Functional screen identifies kinases driving prostate cancer visceral and bone metastasis. Proc Natl Acad Sci USA.

[99] Choi EB, Yang AY, Kim SC, Lee J, Choi JK, Choi C (2016). PARP1 enhances lung adenocarcinoma metastasis by novel mechanisms independent of DNA repair. Oncogene.

[100] Chen Q, Boire A, Jin X, Valiente M, Er EE, Lopez-Soto A (2016). Carcinoma-astrocyte gap junctions promote brain metastasis by cGAMP transfer. Nature.

[101] Weissmueller S, Manchado E, Saborowski M, Morris JPt, Wagenblast E, Davis CA (2014). Mutant p53 drives pancreatic cancer metastasis through cell-autonomous PDGF receptor beta signaling. Cell.

[102] Cueni LN, Hegyi I, Shin JW, Albinger-Hegyi A, Gruber S, Kunstfeld R (2010). Tumor lymphangiogenesis and metastasis to lymph nodes induced by cancer cell expression of podoplanin. Am J Pathol.

[103] Roy SS, Gonugunta VK, Bandyopadhyay A, Rao MK, Goodall GJ, Sun LZ (2014). Significance of PELP1/HDAC2/miR-200 regulatory network in EMT and metastasis of breast cancer. Oncogene.

[104] Samanta D, Park Y, Andrabi SA, Shelton LM, Gilkes DM, Semenza GL (2016). PHGDH expression is required for mitochondrial redox homeostasis, breast cancer stem cell maintenance, and lung metastasis. Cancer Res.

[105] De Semir D, Nosrati M, Bezrookove V, Dar AA, Federman S, Bienvenu G (2012). Pleckstrin homology domain-interacting protein (PHIP) as a marker and mediator of melanoma metastasis. Proc Natl Acad Sci USA.

[106] Bao YN, Cao X, Luo DH, Sun R, Peng LX, Wang L (2014). Urokinase-type plasminogen activator receptor signaling is critical in nasopharyngeal carcinoma cell growth and metastasis. Cell Cycle.

[107] Zhu M, Fejzo MS, Anderson L, Dering J, Ginther C, Ramos L (2010). Periostin promotes ovarian cancer angiogenesis and metastasis. Gynecol Oncol.

[108] Zhang M, Dai C, Zhu H, Chen S, Wu Y, Li Q (2011). Cyclophilin A promotes human hepatocellular carcinoma cell metastasis via regulation of MMP3 and MMP9. Mol Cell Biochem.

[109] Takano S, Reichert M, Bakir B, Das KK, Nishida T, Miyazaki M (2016). Prrx1 isoform switching regulates pancreatic cancer invasion and metastatic colonization. Genes Dev.

[110] Song ZB, Ni JS, Wu P, Bao YL, Liu T, Li M (2015). Testes-specific protease 50 promotes cell invasion and metastasis by increasing NF-kappaB-dependent matrix metalloproteinase-9 expression. Cell Death Dis.

[111] Liao YC, Ruan JW, Lua I, Li MH, Chen WL, Wang JR (2012). Overexpressed hPTTG1 promotes breast cancer cell invasion and metastasis by regulating GEF-H1/RhoA signalling. Oncogene.

[112] Chen DL, Wang DS, Wu WJ, Zeng ZL, Luo HY, Qiu MZ (2013). Overexpression of paxillin induced by miR-137 suppression promotes tumor progression and metastasis in colorectal cancer. Carcinogenesis.

[113] Wang T, Gilkes DM, Takano N, Xiang L, Luo W, Bishop CJ (2014). Hypoxia-inducible factors and RAB22A mediate formation of microvesicles that stimulate breast cancer invasion and metastasis. Proc Natl Acad Sci USA.

[114] Teng Y, Qin H, Bahassan A, Bendzunas NG, Kennedy EJ, Cowell JK (2016). The WASF3-NCKAP1-CYFIP1 complex is essential for breast cancer metastasis. Cancer Res.

[115] O’Connell MP, Fiori JL, Xu M, Carter AD, Frank BP, Camilli TC (2010). The orphan tyrosine kinase receptor, ROR2, mediates Wnt5A signaling in metastatic melanoma. Oncogene.

[116] Mora N, Rosales R, Rosales C (2007). R-Ras promotes metastasis of cervical cancer epithelial cells. Cancer Immunol Immunother.

[117] Whittle MC, Izeradjene K, Rani PG, Feng L, Carlson MA, DelGiorno KE (2015). RUNX3 controls a metastatic switch in pancreatic ductal adenocarcinoma. Cell.

[118] Dahlmann M, Kobelt D, Walther W, Mudduluru G, Stein U (2016). S100A4 in cancer metastasis: Wnt signaling-driven interventions for metastasis restriction. Cancers (Basel)..

[119] Jiang L, Lai YK, Zhang J, Wang H, Lin MC, He ML (2011). Targeting S100P inhibits colon cancer growth and metastasis by Lentivirus-mediated RNA interference and proteomic analysis. Mol Med.

[120] Luchino J, Hocine M, Amoureux MC, Gibert B, Bernet A, Royet A (2013). Semaphorin 3E suppresses tumor cell death triggered by the plexin D1 dependence receptor in metastatic breast cancers. Cancer Cell.

[121] Kaur A, Webster MR, Marchbank K, Behera R, Ndoye A, Kugel CH (2016). sFRP2 in the aged microenvironment drives melanoma metastasis and therapy resistance. Nature.

[122] Wang CA, Drasin D, Pham C, Jedlicka P, Zaberezhnyy V, Guney M (2014). Homeoprotein Six2 promotes breast cancer metastasis via transcriptional and epigenetic control of E-cadherin expression. Cancer Res.

[123] Yanagawa J, Walser TC, Zhu LX, Hong L, Fishbein MC, Mah V (2009). Snail promotes CXCR2 ligand-dependent tumor progression in non-small cell lung carcinoma. Clin Cancer Res.

[124] Ding X, Park SI, McCauley LK, Wang CY (2013). Signaling between transforming growth factor beta (TGF-beta) and transcription factor SNAI2 represses expression of microRNA miR-203 to promote epithelial-mesenchymal transition and tumor metastasis. J Biol Chem.

[125] Huang W, Chen Z, Shang X, Tian D, Wang D, Wu K (2015). Sox12, a direct target of FoxQ1, promotes hepatocellular carcinoma metastasis through up-regulating Twist1 and FGFBP1. Hepatology.

[126] Zhou Y, Wang X, Huang Y, Chen Y, Zhao G, Yao Q (2015). Down-regulated SOX4 expression suppresses cell proliferation, metastasis and induces apoptosis in Xuanwei female lung cancer patients. J Cell Biochem.

[127] Tiwari R, Pandey SK, Goel S, Bhatia V, Shukla S, Jing X (2015). SPINK1 promotes colorectal cancer progression by downregulating Metallothioneins expression. Oncogenesis..

[128] Schmid F, Wang Q, Huska MR, Andrade-Navarro MA, Lemm M, Fichtner I (2016). SPON2, a newly identified target gene of MACC1, drives colorectal cancer metastasis in mice and is prognostic for colorectal cancer patient survival. Oncogene.

[129] Mi Z, Bhattacharya SD, Kim VM, Guo H, Talbot LJ, Kuo PC (2011). Osteopontin promotes CCL5-mesenchymal stromal cell-mediated breast cancer metastasis. Carcinogenesis.

[130] Ke L, Xiang Y, Guo X, Lu J, Xia W, Yu Y (2016). c-Src activation promotes nasopharyngeal carcinoma metastasis by inducing the epithelial-mesenchymal transition via PI3 K/Akt signaling pathway: a new and promising target for NPC. Oncotarget..

[131] Li XJ, Ong CK, Cao Y, Xiang YQ, Shao JY, Ooi A (2011). Serglycin is a theranostic target in nasopharyngeal carcinoma that promotes metastasis. Cancer Res.

[132] van Roosmalen W, Le Devedec SE, Golani O, Smid M, Pulyakhina I, Timmermans AM (2015). Tumor cell migration screen identifies SRPK1 as breast cancer metastasis determinant. J Clin Investig..

[133] Trerotola M, Jernigan DL, Liu Q, Siddiqui J, Fatatis A, Languino LR (2013). Trop-2 promotes prostate cancer metastasis by modulating beta(1) integrin functions. Cancer Res.

[134] D’Amato NC, Rogers TJ, Gordon MA, Greene LI, Cochrane DR, Spoelstra NS (2015). A TDO2-AhR signaling axis facilitates anoikis resistance and metastasis in triple-negative breast cancer. Cancer Res.

[135] Bourcy M, Suarez-Carmona M, Lambert J, Francart ME, Schroeder H, Delierneux C (2016). Tissue factor induced by epithelial-mesenchymal transition triggers a procoagulant state that drives metastasis of circulating tumor cells. Cancer Res.

[136] Ma C, Rong Y, Radiloff DR, Datto MB, Centeno B, Bao S (2008). Extracellular matrix protein betaig-h3/TGFBI promotes metastasis of colon cancer by enhancing cell extravasation. Genes Dev.

[137] Chen J, Chen Z, Chen M, Li D, Li Z, Xiong Y (2009). Role of fibrillar Tenascin-C in metastatic pancreatic cancer. Int J Oncol.

[138] Trauzold A, Siegmund D, Schniewind B, Sipos B, Egberts J, Zorenkov D (2006). TRAIL promotes metastasis of human pancreatic ductal adenocarcinoma. Oncogene.

[139] Xu SH, Huang JZ, Xu ML, Yu G, Yin XF, Chen D (2015). ACK1 promotes gastric cancer epithelial-mesenchymal transition and metastasis through AKT-POU2F1-ECD signalling. J Pathol..

[140] Steder M, Alla V, Meier C, Spitschak A, Pahnke J, Furst K (2013). DNp73 exerts function in metastasis initiation by disconnecting the inhibitory role of EPLIN on IGF1R-AKT/STAT3 signaling. Cancer Cell.

[141] Wu Z, Wei D, Gao W, Xu Y, Hu Z, Ma Z (2015). TPO-Induced metabolic reprogramming drives liver metastasis of colorectal cancer CD110 + tumor-initiating cells. Cell Stem Cell.

[142] Addison JB, Koontz C, Fugett JH, Creighton CJ, Chen D, Farrugia MK (2015). KAP1 promotes proliferation and metastatic progression of breast cancer cells. Cancer Res.

[143] Yang J, Mani SA, Donaher JL, Ramaswamy S, Itzykson RA, Come C (2004). Twist, a master regulator of morphogenesis, plays an essential role in tumor metastasis. Cell.

[144] Wu X, Zhang W, Font-Burgada J, Palmer T, Hamil AS, Biswas SK (2014). Ubiquitin-conjugating enzyme Ubc13 controls breast cancer metastasis through a TAK1-p38 MAP kinase cascade. Proc Natl Acad Sci USA.

[145] Razidlo GL, Magnine C, Sletten AC, Hurley RM, Almada LL, Fernandez-Zapico ME (2015). Targeting pancreatic cancer metastasis by inhibition of Vav1, a driver of tumor cell invasion. Cancer Res.

[146] Yang X, Zhang Y, Hosaka K, Andersson P, Wang J, Tholander F (2015). VEGF-B promotes cancer metastasis through a VEGF-A-independent mechanism and serves as a marker of poor prognosis for cancer patients. Proc Natl Acad Sci USA.

[147] Zelenko Z, Gallagher EJ, Tobin-Hess A, Belardi V, Rostoker R, Blank J (2016). Silencing vimentin expression decreases pulmonary metastases in a pre-diabetic mouse model of mammary tumor progression. Oncogene.

[148] Teng Y, Ren MQ, Cheney R, Sharma S, Cowell JK (2010). Inactivation of the WASF3 gene in prostate cancer cells leads to suppression of tumorigenicity and metastases. Br J Cancer.

[149] Qin L, Yin YT, Zheng FJ, Peng LX, Yang CF, Bao YN (2015). WNT5A promotes stemness characteristics in nasopharyngeal carcinoma cells leading to metastasis and tumorigenesis. Oncotarget..

[150] Cao J, Wang Y, Dong R, Lin G, Zhang N, Wang J (2015). Hypoxia-Induced WSB1 promotes the metastatic potential of osteosarcoma cells. Cancer Res.

[151] El-Naggar AM, Veinotte CJ, Cheng H, Grunewald TG, Negri GL, Somasekharan SP (2015). Translational activation of HIF1alpha by YB-1 promotes sarcoma metastasis. Cancer Cell.

[152] Si W, Huang W, Zheng Y, Yang Y, Liu X, Shan L (2015). Dysfunction of the reciprocal feedback loop between GATA3- and ZEB2-nucleated repression programs contributes to breast cancer metastasis. Cancer Cell.

